# MRBSChain a novel scalable medical records binance smart chain framework enabling a paradigm shift in medical records management

**DOI:** 10.1038/s41598-022-22569-3

**Published:** 2022-10-21

**Authors:** Suhasini Monga, Dilbag Singh

**Affiliations:** Department of Computer Science and Engineering, Chaudhary Devi Lal University, Sirsa, HR 125055 India

**Keywords:** Health policy, Information technology, Computer science, Software

## Abstract

Medical records management had always been a challenging in healthcare sector. Traditionally, medical records are handled either manually or electronically that are under the stewardship of hospitals/healthcare institutions. A patient centric approach is the new paradigm where patient is an inherent part of the healthcare ecosystem controlling the access and sharing of his/her personal medical care information. Medical care information requires robust security and privacy. Also there are other issues like confidentiality, interoperability, scalability, cost efficiency and timeliness that need to be addressed. To achieve these objectives, this paper proposes a novel-scalable patient centric yet privacy preserving framework for efficient and secure electronic medical records management. In addition, proposed system generates a unified trusted record and authentication role mapping for enforcing secure access control for medical records using complex encryption algorithms. This paper identifies 13 key performance factors for performance comparison of proposed framework with traditional models. Ethereum and Binance Smart Chain acted as a benchmark platform for performance evaluation of MRBSChain on the basis of three metrics (transaction cost, average block time and deployment cost).At last, a comparative analysis of MRBSChain with other state of art blockchain systems on the basis of execution time is presented in the paper.

## Introduction

Medical Records Management includes storage and analysis of the patient's health information to track the reasons for infections proficiently, fabricate powerful drugs, effective medical care and lay out an exact avoidance plan^1^. Medical care information sharing is one fundamental step to make medical services more intelligent as medical records are an important source of healthcare knowledge. Medical records management has been viewed as a basic job to improve healthcare knowledge, quality, patient experience and related costs. Sharing of medical services information will assist us in becoming more astute, for example, better comprehension of examples and patterns in general wellbeing, guarantee better quality consideration, better recommendations for practicing or specialists, and plan administrations that make the best of restricted national medical care administration budgets for the wellbeing and prosperity of society^2^. Depending on the infrastructure and cost constraints, various medical record management models are used by different private and government hospitals, clinics and healthcare institutions. Most popular medical record management models are:

- Manual Medical Records (MMR): Manual creation and managing of medical records. Mostly in personal clinics and small hospitals.
- Electronic Medical Records (EMR): Records are created on an in-house computer in private hospitals and a manual copy is assigned to the patient
- Centralized Server Electronic Medical Records (CS-EMR): A client/server model is followed for creating, storing and updating data. Records are managed on central computer that acts as a server internal to the hospital. Multispecialty hospitals and large healthcare institutions use this kind of medical record management model.

**Figure 1:**
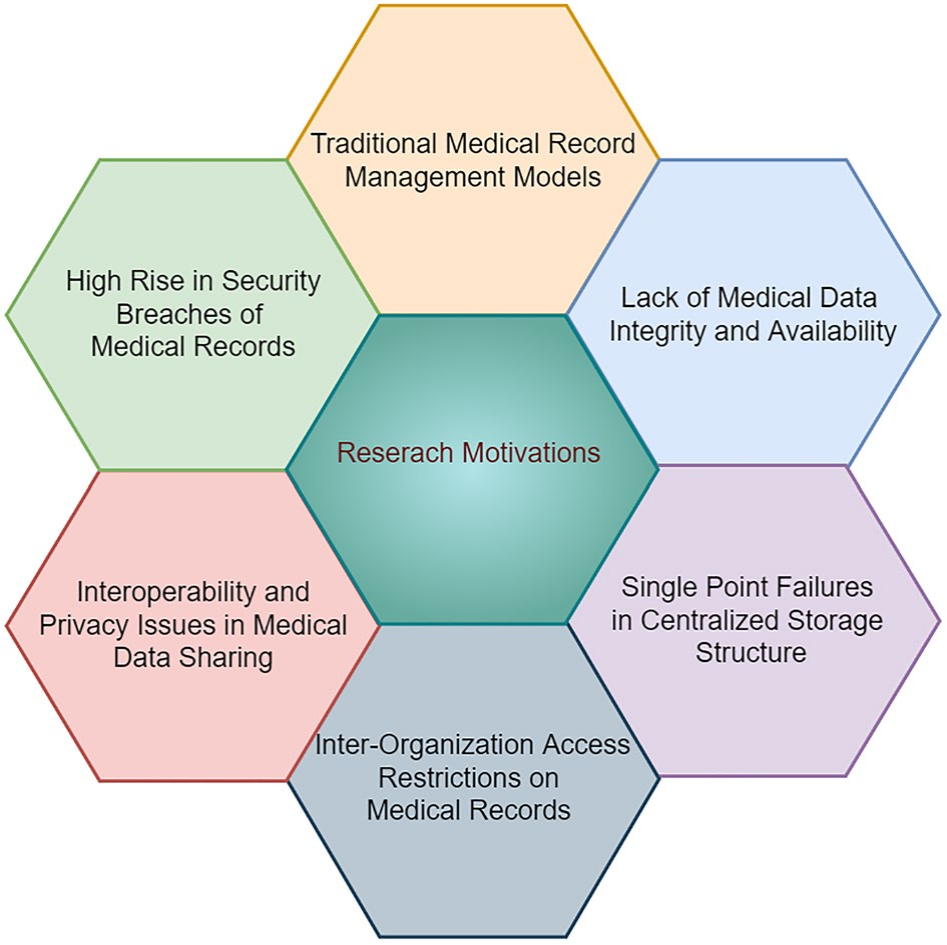
Research motivations.

All these records are managed and access controlled by healthcare providers posing a challenge towards security of patient’s sensitive data. These models also suffers from major security and privacy risks, keeping information up to date, distributed access of a unified copy of trusted medical record, interoperability and scalability^3^. Figure 1 portrays the challenges and issues prevailing in the current systems those acted as research motivations for this study to be conducted.

With the advancement in technology and digitalization it is imperative to improvise medical care systems bridging the gap between patients and healthcare providers. An arising innovation Blockchain has seized curiosity of many researchers in modern and scholastic exploration. Blockchain generates a trusted and permanent record within a circulated decentralized framework, where every hub holds something similar with an equivalent proportion of assurance. Patient’s medical records are very delicate that must be shared securely and accessed by authenticated stakeholders only. The current medical care framework must have a paradigm shift from establishment-driven to patient-driven access control that is private and interoperable^4^.

## Contributions

On the basis of study and analysis, following are the major contributions of this paper:

- A Binance Smart Chain based novel-scalable framework has been developed and the framework entitled as MRBSChain. The proposed framework will assist the hospitals and medical practitioners in the management of medical records that result into speedy data processing to help in early diagnosis of critical patients. The Proposed System also ensures security, privacy, confidentiality, scalability, interoperability, authentication, cost efficiency and unified trusted record. Proposed Decentralized MRBSChain Framework alleviates the issues and challenges existing in the current systems.
- Patient’s privacy has been ensured in the system by implementing a privacy preserving model that enhances the encryption level of medical records by using Block Wallet Address (BW_A_) and Private Hash Key (P_K_). In addition, each record also has its own unique SHA256 Hash Key (SH_K_) which is inherently most complex and Global Unique Identity (gUId).
- Secured access and control of medical records are of utmost importance. To achieve this objective, an Authentication Role Mapping (ARM) is designed among entities: patients, administrators and doctors using Keccak256 Hash Role (KH_R_). This mapping process avoids unauthorized access and data tampering, it also ensures a unified trusted medical record.
- Performance of the proposed framework has been compared and analyzed with traditional medical record management models.13 Key Performance Factors (KPFs) have been identified for this analysis. These KPFs checks the integrity of the proposed work and its importance for the current medical systems.
- Evaluation of the proposed framework is conducted using three important metrics: Transaction Cost (TC), Average Block Time (ABT) and Deployment Cost (DC). Also MRBSChain is compared with other state of art blockchain based systems in terms of Execution Time (ET).

## Literature review

Hao et al. presented a framework for controlling access based on smart contracts to implement resource access control in a trustworthy, auditable and scalable manner by the owner. A contract for access control is implemented on Blockchain to control resource access rules based on attributes flexibility and credibility in access determinations for clients. Owner signs a group of attributes that are given to the clients as off-chain signatures. A test prototype is developed on the Ethereum network and conducted theoretical and experimental analyses for checking its efficiency and scalability^5^.

Hao et al. proposed a protected plan for information sharing giving a client a privileged access in cloud-based IoMT climate. Cloud Sever is empowered re-encrypt the cipher text by re encryption strategies and using ABE as the essential structure block. Patients can easily manage their rights and proficiently share their information. Author evaluated the performance to show the security and productivity of the proposed work^6^.

Ismail et al. evaluated the performance of client/server ideal model in comparison to the blockchain. The outcomes uncover that striking exhibition would be able be accomplished utilizing blockchain. Moreover, the unchanging and legitimate patients' information in the blockchain can help united wellbeing experts in better visualization and finding support through AI and man-made consciousness. Also, proposed a basic design of a blockchain based system for medical services that provides usefulness to medical services exchanges^1^.

Hathaliya et al. talked about advances of healthcare 1.0 towards healthcare 4.0. An in-secure medical care record framework might incite the clinical consideration data break where software engineers can get full permission to patients' email records, messages, and reports whereas protected and a safe framework can give satisfaction to patients and different elements. This paper presents an exhaustive report and assessment of state of the art suggestions to stay aware of safety and assurance in medical care. The advantages and cutoff points of various security and assurance strategies are inspected in the paper^7^.

Murugan et al. analyzed the issues of cloud based storage of medical records and proposed a blockchain based prototype Health Information Exchange (HIE).Hyperledger Fabric is utilized to hold the fundamental security expected in the proposed framework. Hyperledger fabric is a permissioned network where the proprietor of the organization holds all the entrance privileges^8^.

Nguyen et al*.* proposed EHRs sharing structure that joins blockchain and IPFS on portable cloud storage. Prototype is built on Ethereum and Amazon Cloud. Smart contracts are utilized to accomplish secure EHRs dividing between various patients and clinical suppliers. The framework assessment and security investigation additionally illustrate execution upgrades in lightweight access control plan, security and information protection levels, contrasted with existing information sharing models^9^.

Hasselgrena et al. focused to methodically audit, survey and blend peer-inspected publications using/proposing to use blockchain to further develop cycles and administrations in medical care, wellbeing sciences and medical knowledge. The outcome demonstrates that Electronic Health Records and Individual Health Records are the most designated regions utilizing blockchain innovation. According to the study Ethereum and Hyperledger fabric appear to be the most utilized stages/systems in this area^10^.

After the in depth review of related work, it is clear that there is a need of blockchain innovation that not only can benefit financial sectors but it can be applied to other areas like healthcare, insurance, supply chain etc. Blockchain being decentralized, immutable and robust in nature can provide immense benefits to the medical services domain and society at large. It can resolve all the existing issues and challenges of the healthcare industry providing better patient care and medical record management. This paper gives a novel blockchain contribution in the research area of healthcare information technology.

## Background

Since decentralization is the need of the hour, all gratitude goes to the well-known paper of Satoshi Nakamoto in 2008, presenting Bitcoin and blockchain innovation^11^. Blockchain may be presumed as a decentralized record ledger where the each transaction is executed to form a block and each block after validation is appended to the chain forming a structure called blockchain. Blockchain is empowered by joining of many advance technologies like Cryptographic hash, computerized signature and consensus determination^12^. Due to diverse application areas different blockchains has been introduced in the world of technology in the past few years. This paper focuses on Ethereum and Binance Smart Chain.

### Ethereum

Ethereum addresses a blockchain with an underlying Turing complete programming language. Smart Contracts includes a bunch of cryptographic guidelines that are executed provided that specific circumstances are met^13^. Each hub in the Ethereum network runs under Ethereum Virtual Machine (EVM). The code for the smart contracts is written in Solidity that is the most popular programming language for writing code. The smart contracts are converted into EVM code and afterward executed by the hubs. An Ethereum block contains list of transactions and recent state along with block number, nonce, difficulty etc. A new state is created by referring to the previous state every time a new transaction enters into the transaction list^11^. Ethereum Block Time is usually between ~ 7 and 15 s for execution and creation of block. Ethereum currently uses Proof of Work as the underlying algorithm for consensus determination. In Proof of Work algorithm public miners or block validators have to go through an extraordinary race of experimentation of solving puzzles to track down the nonce for a block. A new block will be added to the blockchain only if it has a valid nonce^11^.

### Binance Smart Chain

Although Ethereum was introduced to resolve many issues of Bitcoin-First Blockchain yet it suffers from the issues of Block Time, Scalability and Cost Issues. Binance Smart Chain(BSC)was introduced in 2020 after three years of launch of Binance Chain in 2017 by Chapeng Zhao Yi He^14^ removing the barriers of Ethereum. BSC is the recent development in Blockchain technology that is parallel to Binance Chain and compatible to Ethereum Virtual Machine (EVM). BSC is the disruptive architecture that has smart contract capabilities, very high throughput, less cost, less block time and more scalability. BSC being a parallel blockchain to Binance Chain has no scalability issues like Ethereum which is a single chain^14^. BSC follows a strong hybrid consensus algorithm Proof of Stacked Authority, a combination of Proof of Stake and Proof of Authority. BSC validators are members who "stake" a specific number of Binance Coin (BNB). The number of dynamic validators is restricted to 21 and just dynamic validators are qualified to approve transactions. The main 21 validators with the biggest number of BNB become dynamic block validators in the blockchain network^14^. Therefore in comparison to Ethereum; BSC can be applied as a better decentralized and more scalable approach for medical records management in modern healthcare. Table 1 represents the comparison between Ethereum and Binance Smart Chain.

**Table 1:** Comparison: Ethereum versus Binance Smart Chain.

Features	Ethereum	Binance Smart Chain
Launch	2015	2020
Consensus mechanism	Proof of work	Proof of stacked authority
Chain architecture	Single chain	Dual chain
Number of validators	Thousands	21
Validators selection	Solving puzzles	Taking turns
Block time	~ 7–15 s	~ 3 s
Transaction cost	High	Low
Throughput	Low	High
Scalability	Less	More
Reward token	ERC20	BNB
Validators type	Public	Private

## Proposed framework

This section initially begins with existing centralized EMR framework. The likely constraints of centralized architecture are featured and justification of decentralized blockchain based framework is presented in this section.

### Centralized EMR framework

**Figure 2:**
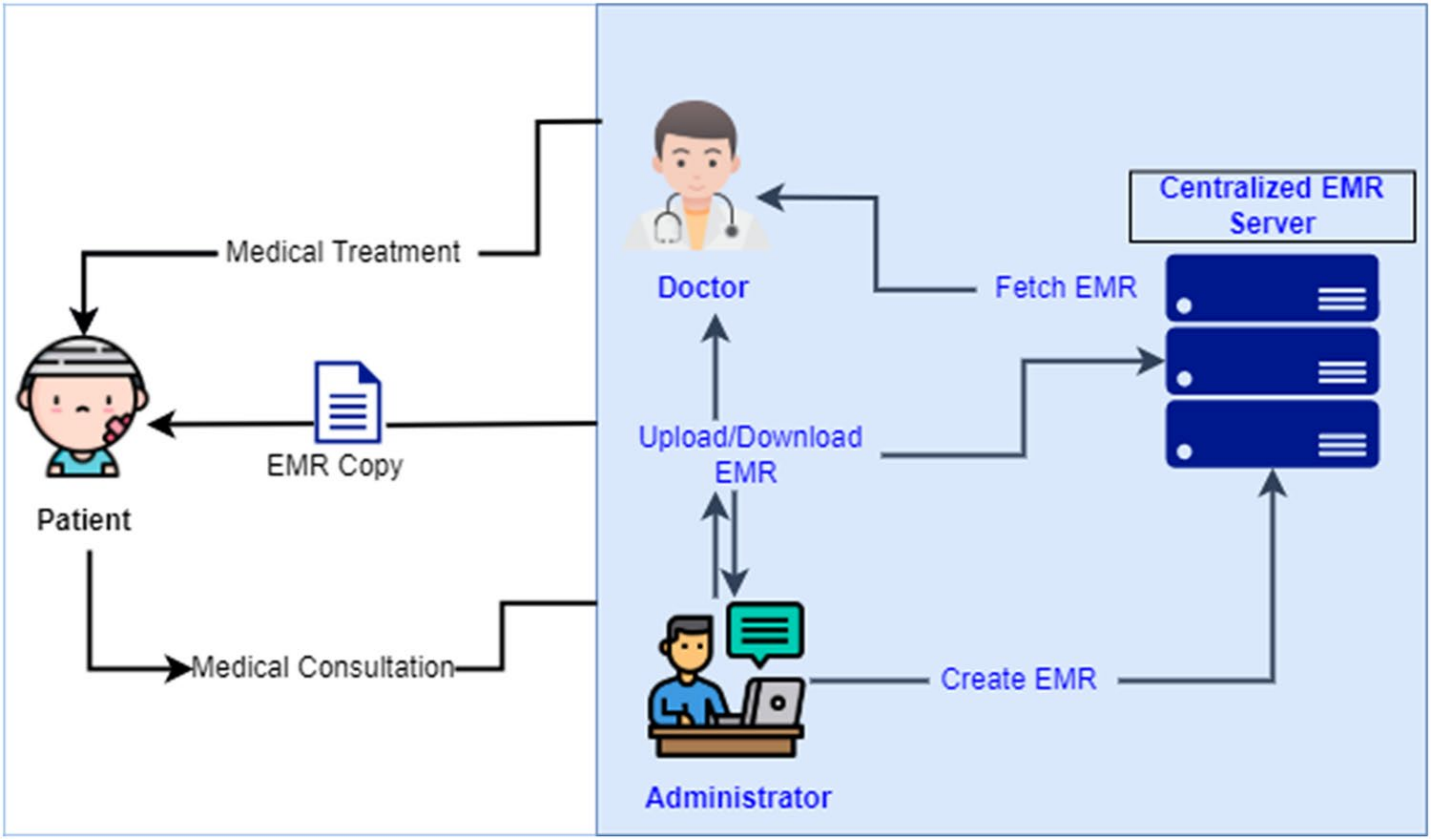
Traditional centralized EMR framework.

Creating, updating and storing of EMRs are generally managed by either doctors or administrators in a centralized database server, internal to the hospitals, which brings an issue that patients leave information dispersed across different hospitals. It is noteworthy that these records are created in local databases of various hospitals after patients visit them. Along these lines, patients lose access to past information regardless of whether it has a place with them. At the point when they visit different hospitals, they do not have access to their past records to give the specialist their definite past prescription records^15,16^. Interoperability, security and privacy challenges between various hospital frameworks present extreme obstacles to information sharing. It is hard for individuals to get the information they need in view of absence of bound together information. Hence; in traditional establishment-centric and centralized approach patients are external entities to the existing EMR system. On the other hand doctors or administrators being internal entities in the system have full access and control over patient's sensitive health medical records. Figure 2 presents traditional centralized EMR framework.

### Proposed decentralized MRBSChain EMR framework

**Figure 3:**
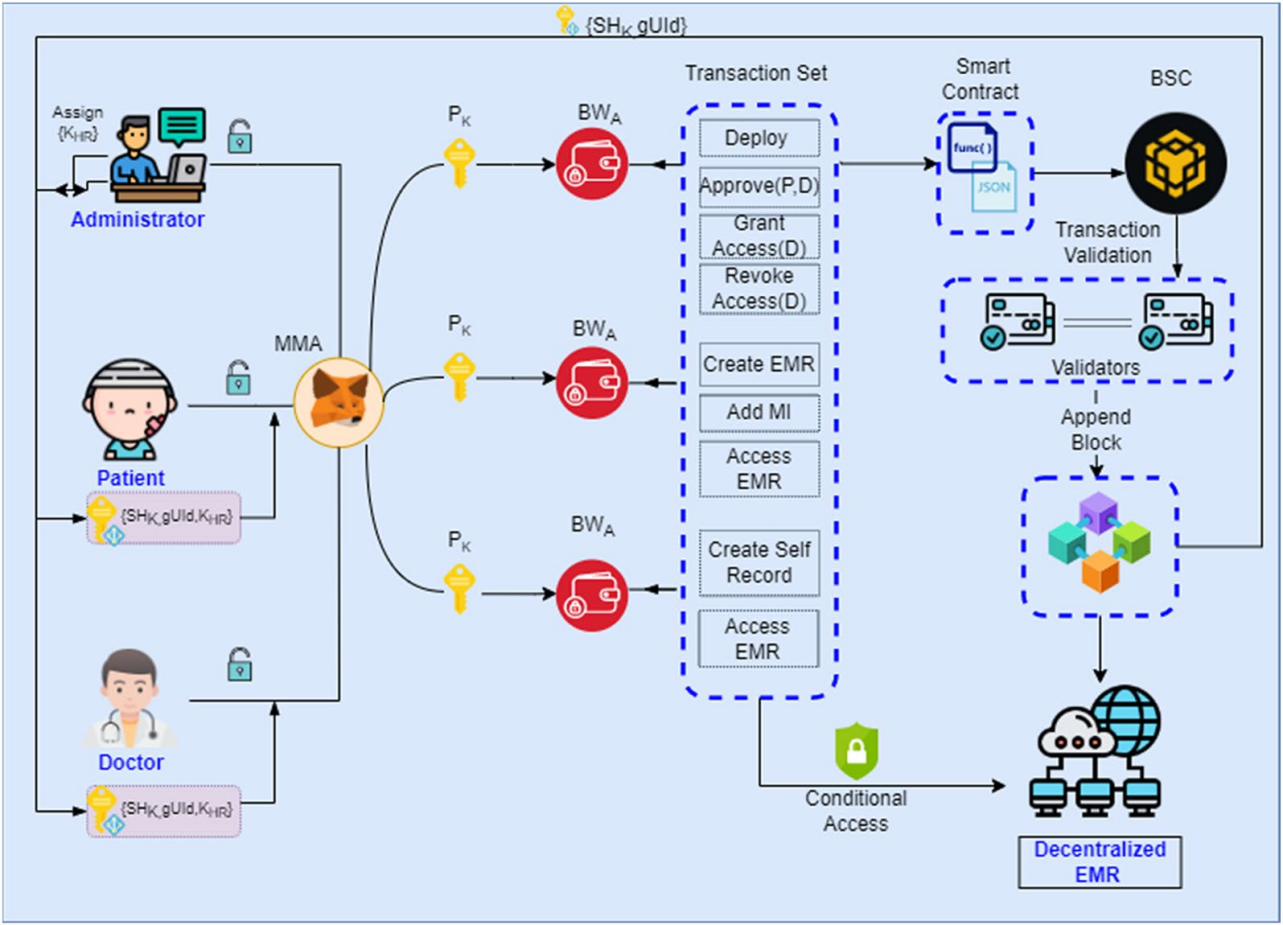
Proposed decentralized MRBSChain EMR framework.

In order to resolve the challenges and issues of traditional centralized EMR frameworks this paper proposes a novel decentralized MRBSChain EMR framework for medical records management as depicted in Fig. 3. Proposed system is designed following a patient centric approach keeping patients, doctors and administrators all as internal entities in blockchain framework. Every new entity in the proposed framework has to undergo different levels of phases that begin with creation of a new block with registration of a new entity in the system. Initially a new entity has a private hash key and unique block wallet address. If the entity is already existing an error is generated otherwise once registered successfully, a new block on the chain is appended with a SHA256 private hash key and global unique identifier. In the next phase every entity is approved and verified by the administrator of the system in case if approval fails, no further access is permitted to the new entity, but once approved administrator assigns a role either Doctor or Patient to the entity. This role is again inherently a private hash key generated using Keccak256 encryption algorithm. Next phase differs for both patients and doctors, as patient can continue adding his medical issues and other details; doctors need to be assigned access to the patient records but it is done by the administrator only. Administrator can only approve and revoke access of the medical record.

#### Privacy preserving identity model

For every new entity, a new Block Wallet Address(BWA) is assigned using Private Hash Key (P_K_) and Metamask Account (MMA) as shown in Eq. (1) where i represents each entity that can vary from i = 1…n.

1 $$\left\{ {{\text{P}}_{{{\text{K}}({\text{i}})}} ,{\text{ MMA}}_{{({\text{i}})}} } \right\} \Rightarrow {\text{BW}}_{{{\text{A}}({\text{i}})}}$$

Each Metamask Account has some BNB tokens in the Metamask wallet. After deployment by the administrator; patient and doctor entity using their Block Wallet Address can register and create their records on the BSC. If the transaction of record creation is successful and validated by the validators on the BSC network, a new block is appended on the BSC.

For each new block, a unique SHA256 private hash key (SH_K_) and global unique identity (gUId) is generated as shown in Eq. (2) where i represents each entity that can vary from i = 1…n.

2 $${\text{BW}}_{{{\text{A}}({\text{i}})}} \Rightarrow \left\{ {{\text{SH}}_{{{\text{K}}\left( {\text{i}} \right),}} {\text{gUId}}_{{({\text{i}})}} } \right\}$$

#### Authentication role mapping (ARM)

To enforce security and access control an improvised consensus is implemented in the proposed work referred in the research as ARM. It is a process of granting authentication roles to patients or doctors and mapping their BW_A_ with each other for secured EMR access. Once a new entity, either patient or doctor successfully registers; a new SHA256 Private Hash Key (SH_K_) and global unique identity (gUId) is assigned as shown in Eq. (2). Every new entity is approved by the administrator, without approval neither patient nor doctor can perform any operation in the system. Once administrator approves every entity is granted a role (Patient/Doctor). This role is unique private hash key that is generated using Keccak256 hashing algorithm and represented as K_HR_ as shown in Eq. (3) where i represents each entity that can vary from i = 1…n.

3 $$\left\{ {{\text{SH}}_{{{\text{K}}\left( {\text{i}} \right),}} {\text{gUId}}_{{({\text{i}})}} } \right\} \Rightarrow {\text{ K}}_{{{\text{HR}}({\text{i}})}}$$

After the approval process, patient entity can add medical issues to the existing record and can view, access his/ her EMR anywhere anytime as they are saved on a decentralized network. Administrator assigns access of the patient records to the doctor for consultation process. Proposed framework internally implements Encrypted Hash Mapping where Block Wallet Addresses (BW_A_) for both Patient and Doctor Entity are mapped as shown in Eq. (4) where i represents each entity that can vary from i = 1…n. For this mapping to be successful, each Patient and Doctor entity must be successfully registered and approved. Each entity must have SHA256 Private Hash Key, Global Unique Identifier, Keccak256 Hash Role (SH_K,_ gUId, K_HR_).

4 $${\text{BW}}_{{\text{A}}} \left( {{\text{Patient}}_{{({\text{i}})}} } \right) \Leftrightarrow {\text{BW}}_{{\text{A}}} \left( {{\text{Doctor}}_{{({\text{i}})}} } \right)\left\{ {{\text{SH}}_{{{\text{K}}\left( {\text{i}} \right),}} {\text{gUId}}_{{({\text{i}})}} ,{\text{K}}_{{{\text{HR}}({\text{i}})}} } \right\}$$

After successful mapping Doctor can conditionally access patient’s EMR only if he/she is assigned to the Patient by the Administrator. For maintaining security and patient’s control over his/her personal records, access is revoked for that particular doctor by the administrator once the purpose is served.

## Implementation

**Figure 4:**
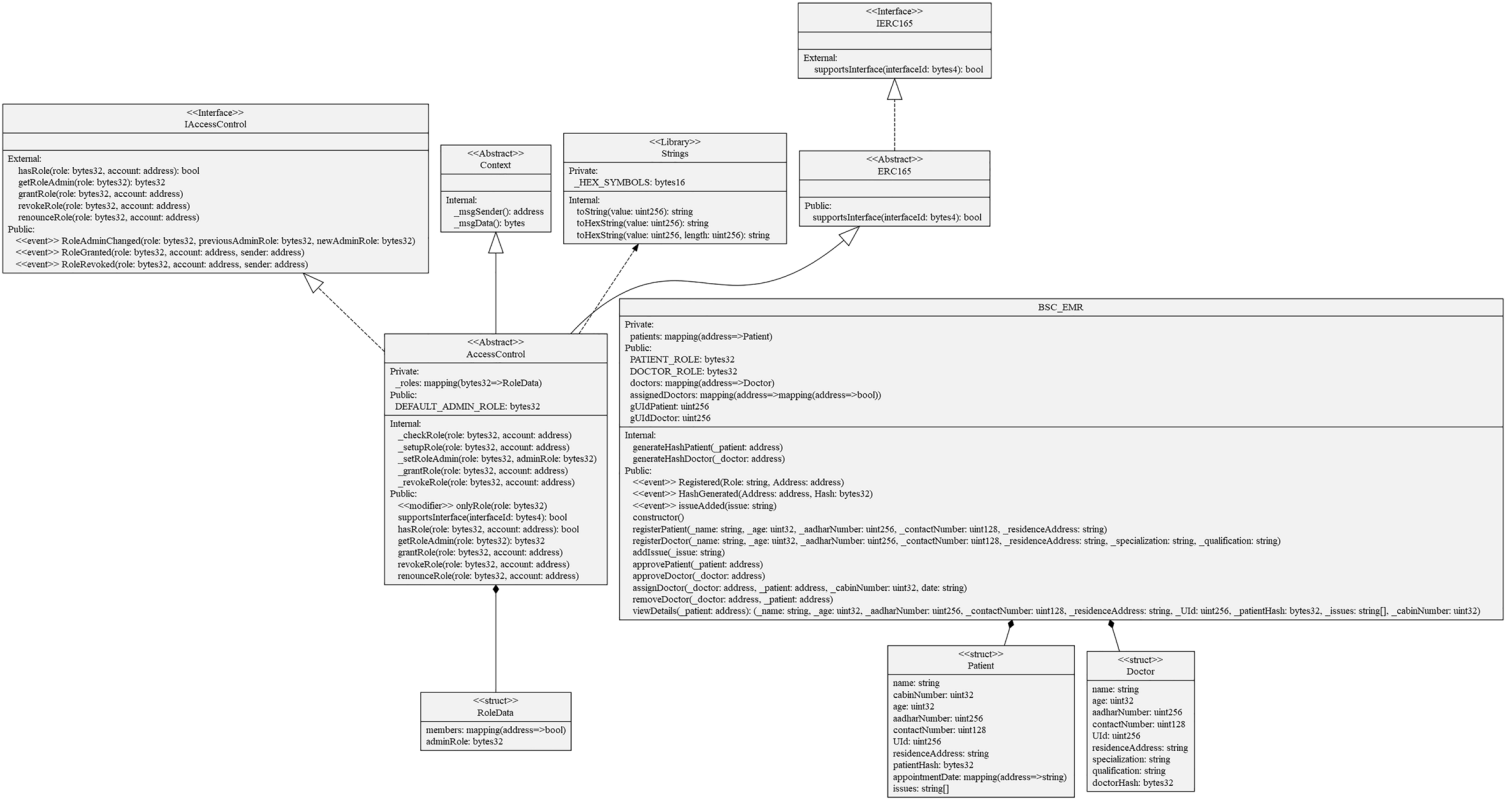
UML diagram: solidity code MRBSChain framework.

The MRBSChain framework has been deployed on BSC test network using Metamask accounts for three major stakeholders in EMR management: Administrator, Patient and Doctor. Smart contract BSC_EMR is created using the programming language Solidity. Since BSC is EVM compatible, platform used for development and coding of smart contracts is remix.ethereum.org. Table 2 gives the details for technical specifications. The entire code structure of the framework is shown through a precise UML Diagram in Fig. 4. UML Diagram of MRBSChain shows all the private, public structures, interfaces, inheritance levels, and events. All internal and external functions that are executed as transactions when deployed on the BSChain network are also listed in the diagram.

**Table 2:** Technical specifications.

Name	Description
Programming language	Solidity
Development platform	Remix.ethereum.org
Blockchain	Binance smart chain
Encryption algorithms	SHA256, Keccak256
Keys	P_K_, BW_A_
Deployment network	BSC test network
Token	BNB
Virtual machine	EVM

Table 3 lists all the constraints that are implemented in the code for valid and invalid transactions. If any constraint is violated, the transaction fails and a message along with transaction unsuccessful is displayed in the output terminal in remix.

**Table 3:** Constraints for testing validity of transactions.

Validation constraints		
Entity type	Successful transaction	Unsuccessful transaction
Administrator	Contract deployed successfully Patient approved Doctor approved Assign doctor to patient–grant access Remove doctor access	Does not have admin rights Patient not registered Doctor not registered Assignee is not a valid doctor The address assigned for is not a valid patient Not authorized to remove doctor The doctor is not assigned to this patient
Patient	Patient registered successfully Patient approved to add medical issues Patient can access records	Already existing patient Not approved to add medical issues
Doctor	Doctor registered successfully Doctor can access records	Already existing doctor Access not granted to view details of this patient

**Figure 5:**
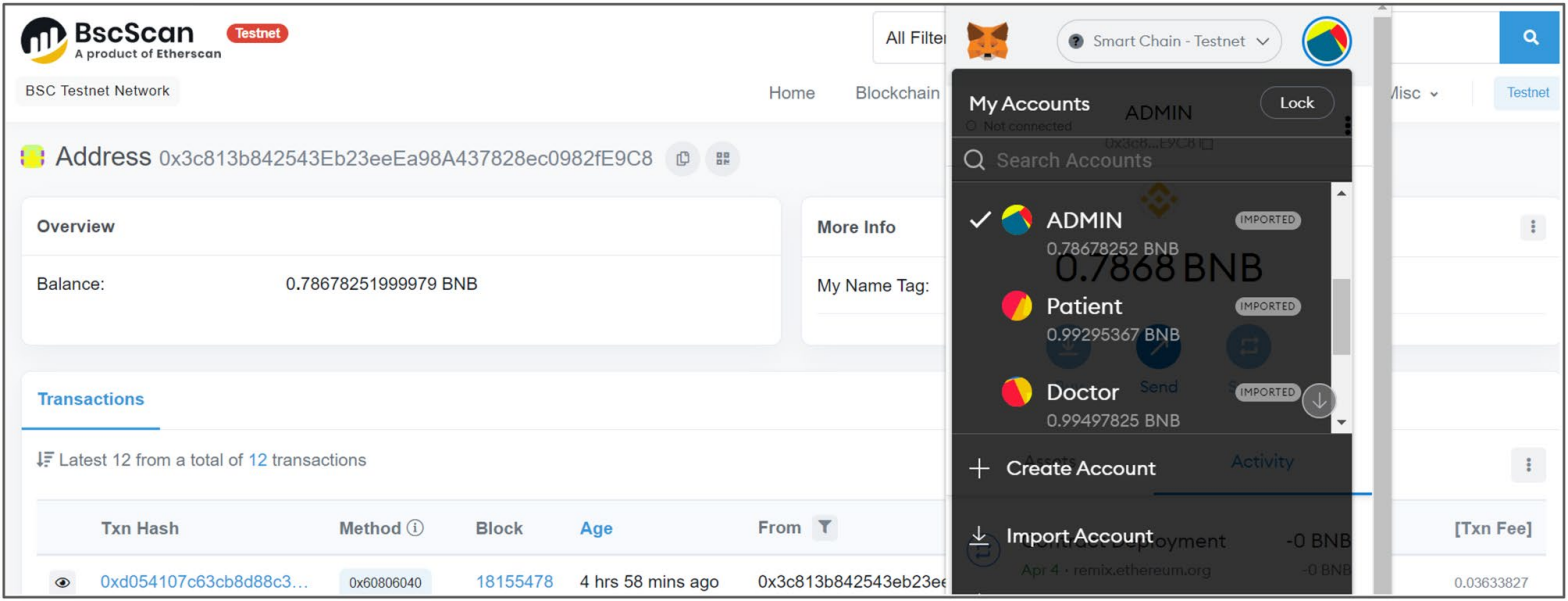
BSC output for admin BW_A_.

**Figure 6:**
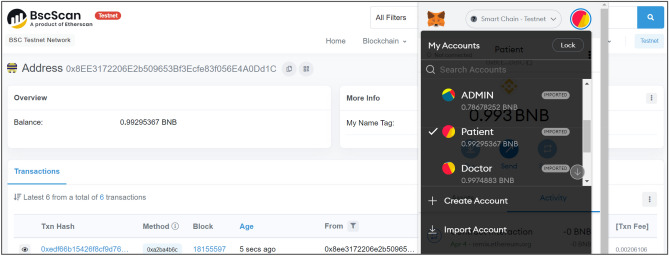
BSC output for patient BW_A_.

**Figure 7:**
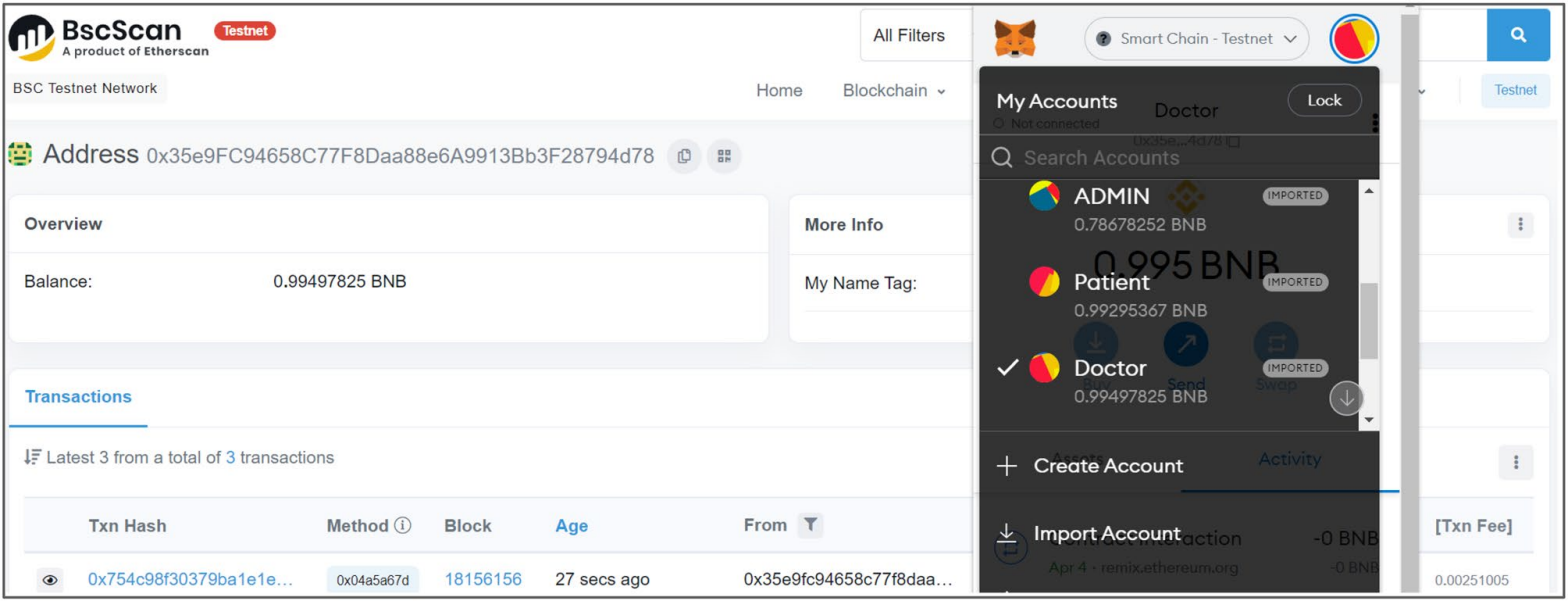
BSC output for doctor BW_A_.

On the other hand when a transaction is successful, its output can be viewed on the output terminal of remix and also on the BSC test network using transaction address or hash generated or BW_A_. For real time deployment of MRBSChain framework, three different Metamask accounts for: Admin, Patient and Doctor have been created using P_K_ and 1 BNB. Each account has a BW_A_ that is used for executing different transactions. It is not feasible to show all the transaction outputs here in the paper, therefore, three successful transaction outputs for contract deployment, patient and doctor registration have been shown in Figs. 5, 6 and 7.

## Performance evaluation

As the proposed MRBSChain is the first EMR framework, that is implemented and deployed on the BSC. The performance of the proposed work has to be evaluated and compared with traditional record management models described in “Contributions” section. The performance of MRBSChain has been measured under three metrics: Transaction Cost (TC), Deployment Cost (DC) and Average Block Time (ABT) with Ethereum Blockchain. In addition to the above traditional models, proposed work has been evaluated with other existing state of art blockchain based EMR systems also.

### Performance comparison with traditional systems

In the first scenario the proposed Binance Smart Chain-Electronic Medical Records (BSC-EMR) is compared with traditional medical records management models that are Manual Medical Records (MMR), Electronic Medical Records (EMR) and Centralized Server-Electronic Medical Records (CS-EMR).

**Figure 8:**
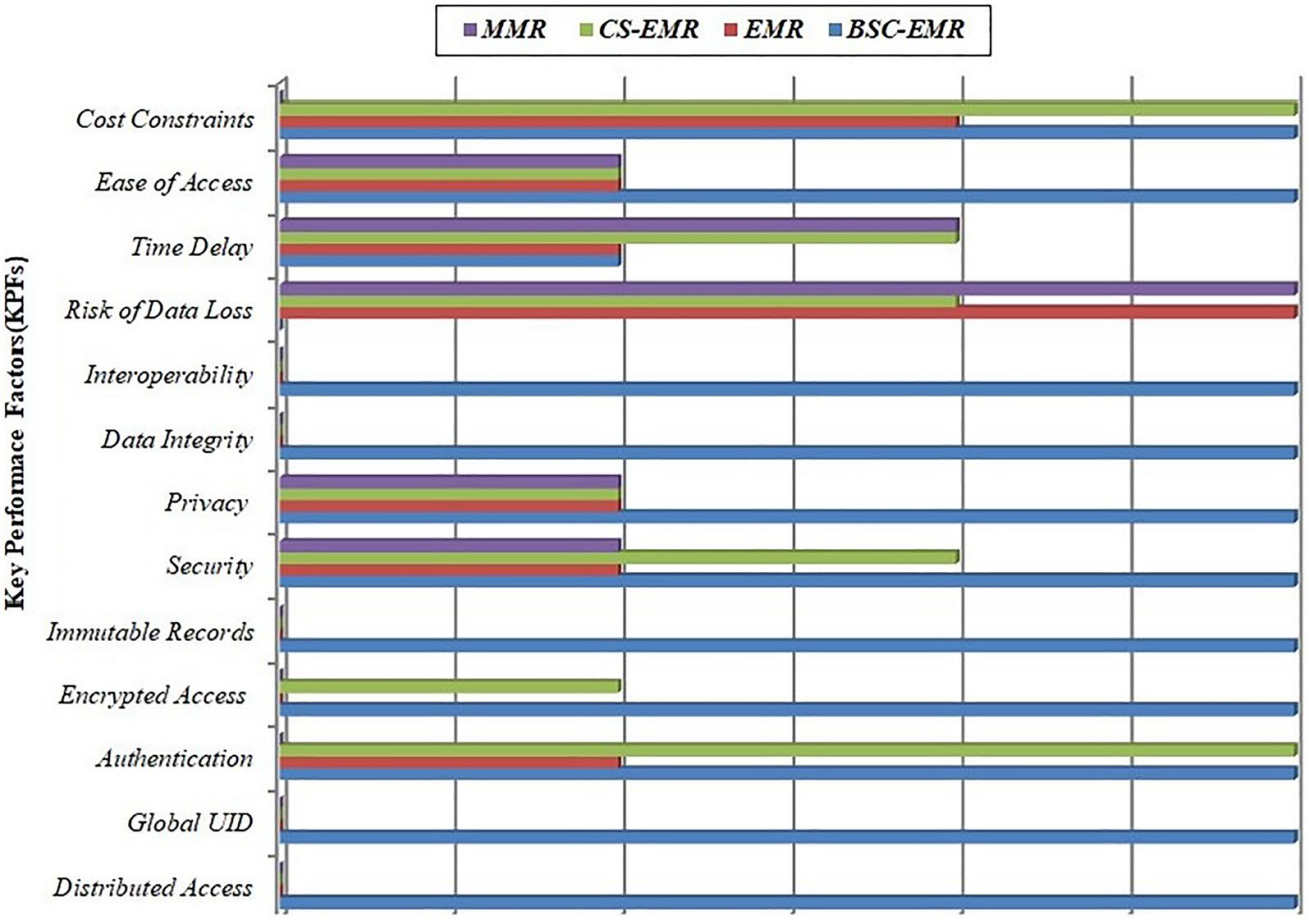
KPFs analysis: MMR, EMR, CS-EMR, and BSC-EMR.

The performance has been compared and analyzed on the basis of 13 Key Performance Factors (KPFs) that has been uniquely identified for this research. These KPFs are the inherent factors including all the challenges and benefits that need to be integrated in medical systems dealing with medical record management of patients. Each KPF has its own importance and defines the necessities of modern healthcare. All the four models (MMR, EMR, CS-EMR and BSC-EMR) been rated for unique Key Performance Factor using 4 metrics: Yes-Y, No-N, High-H, Low-L. The KPF analysis shown in Fig. 8 clearly depicts that the proposed BSC-EMR has integrated maximum KPFs as compared to MMR, EMR and CS-EMR. The 4 metrics have indicator mathematical values used for performance evaluation:

- Yes[3]: Implies full integration of KPF
- High[2]: Implies at most integration of KPF
- Low[1]: Implies at least integration of KPF
- No[0]: Implies no integration of KPF

### Performance comparison: Binance Smart Chain (BSC) versus Ethereum (ETH)

The performance of the proposed work is evaluated in terms of three metrics: Deployment Cost (DC), Average Block Time (ABT) and Transaction Cost (TC). The proposed framework is deployed on both Ethereum and Binance Smart Chain. The deployment server used for ETH is Java Virtual Machine (JVM) using free ether accounts for Administrator, Patient and Doctor. Three real Metamask accounts are created for Administrator, Patient and Doctor having 1 BNB in the Meta mask wallet for deployment on the BSC Test Network.

Every transaction has a cost associated with it in blockchain network. This paper is using gas as the measuring unit for both Deployment Cost (DC) and Transaction Cost (TC).

**Figure 9:**
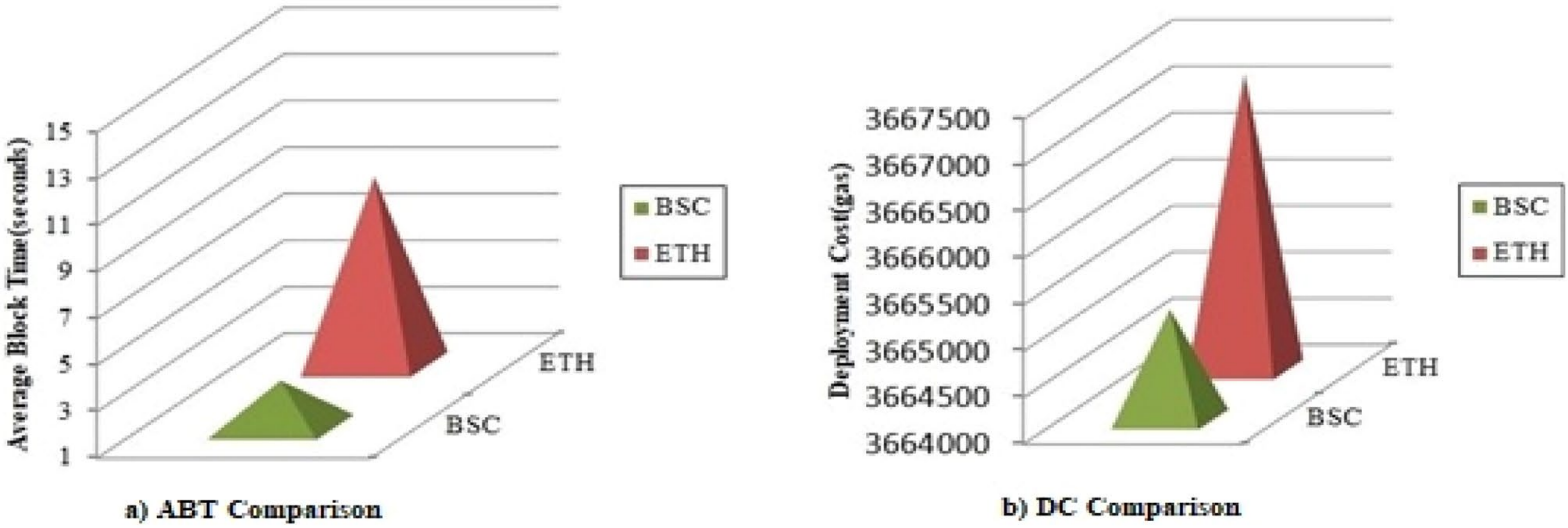
Performance comparison between BSC and ETH: (**a**) ABT comparison, (**b**) DC comparison.

**Figure 10:**
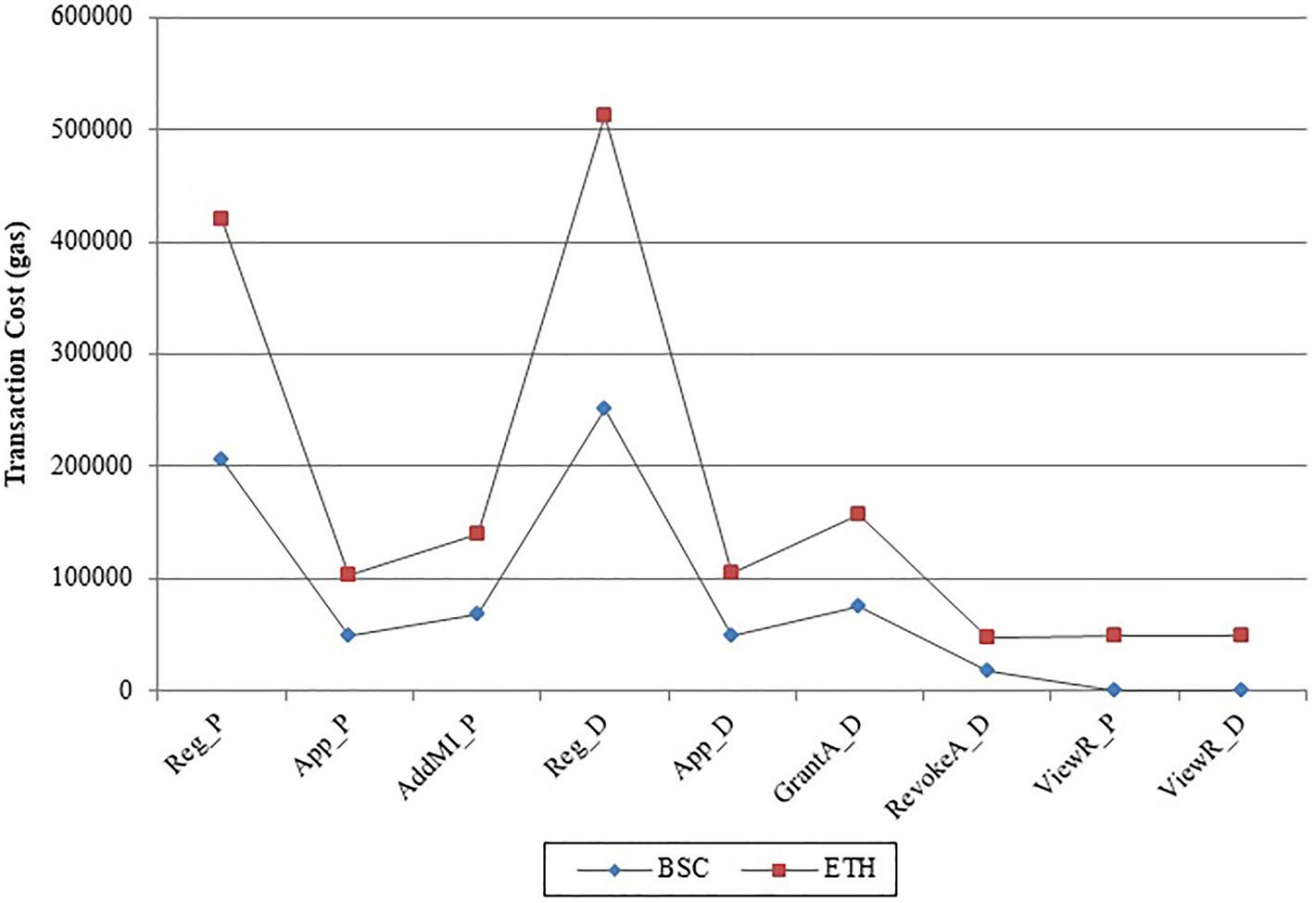
TC comparison of BSC vs. ETH.

Figure 9a ABT Comparison and Fig. 9b DC Comparison shows the Average Block Time (ABT) and Deployment Cost (DC) comparison between BSC and ETH. Figure 10 shows the Transaction Cost (TC) comparison for each transaction in the framework on both BSC and ETH. BSC is a better blockchain platform in comparison to ETH that is clearly indicated in the figures below:

### Performance comparison with other state of art blockchain based EMR systems

**Figure 11:**
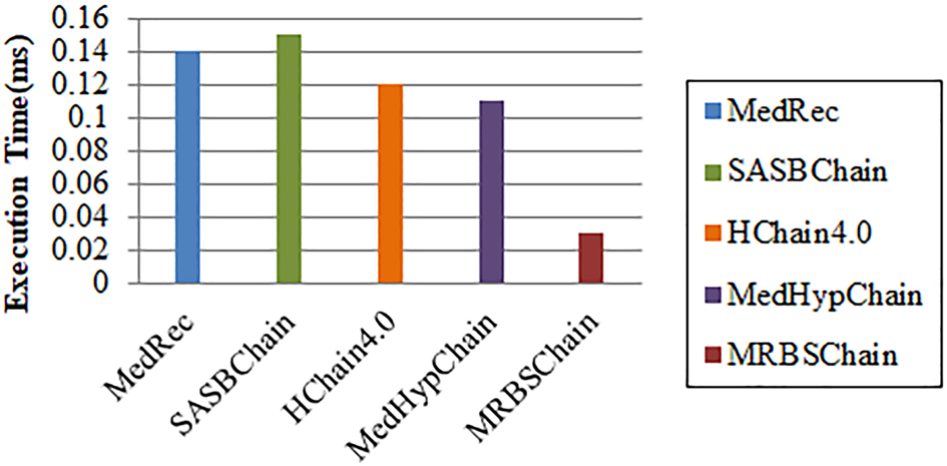
ET comparison of MRBSChain with other blockchain based EMR systems.

The performance of MRBSChain is compared with existing systems: Azaria et al.^17^, Guo et al.^2^, Hathaliya et al.^7^, Kumar et al.^4^ in terms of Execution Time (ms). Since BSC has high throughput and less execution time; it is better as compared to other systems where cost and time constraints are important. Figure 11 shows the ET comparison of MRBSChain with systems mentioned above. Thus, it can be proved that proposed work is efficient in medical records management.

## Conclusion

This paper proposed a novel-scalable blockchain framework for decentralized and secure medical records management. Proposed MRBSChain framework is the first of its kind to be deployed on Binance Smart Chain. In today’s era of digitization a patient centric approach must be designed for secured access of his/her own medical records. Keeping in view this major paradigm shift from establishment-centric to patient-centric system is achieved in this research. MRBSChain as a system guarantees privacy, decentralization, interoperability, adaptability, validation, practicality, cost effectiveness, timeliness and unique trusted copy of patient record. MRBSChain integrates a security saving model utilizing Private Hash Keys (P_K_), and Block Wallet Address (BW_A_) creating SHA256 Hash Key (SH_K_) that is innately most intricate and global unique identity (gUId). An Authentication Role Mapping (ARM) is intended for authorizing security and access control utilizing Keccak256 Hash Role (K_HR_). Performance of MRBSChain is compared, evaluated and analyzed under different real time scenarios and using metrics.

## Data Availability

Data is generated using the proposed framework and is tested for analyzing the performance of proposed work. No predefined data sets are used or gathered in the research. Also the proposed work is tested for different record management models being used in existing systems in hospitals. Due to privacy concerns hospitals are not permitted to disclose their details. The datasets used and/or analyzed during the current study available from the corresponding author on reasonable request.
